# The Effect of Curing Temperature on the Properties of Cement Pastes Modified with TiO_2_ Nanoparticles

**DOI:** 10.3390/ma9110952

**Published:** 2016-11-23

**Authors:** Karine Pimenta Teixeira, Isadora Perdigão Rocha, Leticia De Sá Carneiro, Jessica Flores, Edward A. Dauer, Ali Ghahremaninezhad

**Affiliations:** 1 Department of Civil, Architectural and Environmental Engineering, University of Miami, Coral Gables, FL 33146, USA; karine.pimentateixeira@gmail.com (K.P.T.); isadora.perdigao@hotmail.com (I.P.R.); leticia.carneiro@ufv.br (L.D.S.C.); j.flores7@umiami.edu (J.F.); 2 Department of Biomedical Engineering, University of Miami, Coral Gables, FL 33146, USA; edauer@miami.edu

**Keywords:** hydration, cement paste, elevated temperature, TiO_2_ nanoparticles

## Abstract

This paper investigates the effect of curing temperature on the hydration, microstructure, compressive strength, and transport of cement pastes modified with TiO_2_ nanoparticles. These characteristics of cement pastes were studied using non-evaporable water content measurement, X-ray diffraction (XRD), compressive strength test, electrical resistivity and porosity measurements, and scanning electron microscopy (SEM). It was shown that temperature enhanced the early hydration. The cement pastes cured at elevated temperatures generally showed an increase in compressive strength at an early age compared to the cement paste cured at room temperature, but the strength gain decreased at later ages. The electrical resistivity of the cement pastes cured at elevated temperatures was found to decrease more noticeably at late ages compared to that of the room temperature cured cement paste. SEM examination indicated that hydration product was more uniformly distributed in the microstructure of the cement paste cured at room temperature compared to the cement pastes cured at elevated temperatures. It was observed that high temperature curing decreased the compressive strength and electrical resistivity of the cement pastes at late ages in a more pronounced manner when higher levels of TiO_2_ nanoparticles were added.

## 1. Introduction

Nanomaterials have the potential to revolutionize the construction industry by improving the performance and durability of construction materials, as well as imparting new functionalities to these materials [1,2,3,4]. Nanomaterials such as silica nanoparticles [5,6,7,8,9,10], carbon nanotubes [11,12,13], TiO_2_ nanoparticles [14,15,16,17,18,19,20,21,22,23,24,25], and clay nanoparticles [26,27,28,29] have been studied to investigate their effect on cementitious materials. The use of TiO_2_ nanoparticles in construction materials has been the focus of intense research in the recent years, primarily due to its self-cleaning and air-cleaning properties, arising from their photocatalytic characteristics [30,31]. Lackhoff et al. [14] showed an increase in the compressive strength of mortars containing TiO_2_ nanoparticles. The influence of TiO_2_ nanoparticles on the hydration of cementitious materials was examined by researchers [15,16,17] and shown to enhance hydration due to the high surface area of these nanoparticles, increasing the nucleation sites for the hydration reaction. Lee et al. [18] reported that compressive strength increased by up to 10% TiO_2_ nanoparticle replacement at a low water-to-cement ratio (0.4) and did not show degradation at high water-to-cement ratios (0.6). Zhang et al. [19] demonstrated that TiO_2_ nanoparticles improved the compressive strength and mitigated the drying shrinkage of cementitious materials. In addition, the addition of TiO_2_ nanoparticles has been shown to improve the densification of the microstructure, thereby enhancing the durability of the cementitious materials [20,23,25,32,33].

Due to variability in the environmental temperature during mixing and curing of cementitious materials in practice, it is important to understand how curing temperature influences the behavior of cementitious materials. In addition, high temperature curing is relevant in applications such as oil well cement and precast industry. Prior studies indicated an increase in the early age strength due to enhanced hydration in cementitious materials cured at elevated temperatures, compared to curing at room temperature [34,35,36,37,38]. However, a reduction in the compressive strength of cementitious materials, after the initial enhancement, was observed as a result of curing at elevated temperatures compared to the room temperature curing [34,35,36,37,39]. The effect of curing at high temperatures on the microstructure of cementitious materials was investigated and shown to promote increased porosity and induce heterogeneous distribution of hydration product in the microstructure of the materials [34,36,37,40,41,42]. Although the effects of TiO_2_ nanoparticles and curing temperature have been individually studied in the past, the effect of curing temperature on the behavior of cement paste modified with TiO_2_ nanoparticles has not received attention. In one particular recent study, Jayapalan et al. [43] indicated an increase in the temperature sensitivity of early age hydration of cementitious materials as a result of modification with TiO_2_ nanoparticles. However, the influence of curing temperature on the microstructure and properties of cementitious materials modified with TiO_2_ nanoparticles has not been examined in the past. Knowledge of this influence is important in the engineering of cementitious materials modified with nanoparticles in applications such as oil well cement and precast industry.

Therefore, this paper aims to address this knowledge gap by investigating the hydration, compressive strength, and transport behavior of cement paste modified with TiO_2_ nanoparticles, cured at varied temperatures. The hydration of cement pastes was evaluated using non-evaporable water content measurement. The compressive strength test was performed to investigate compressive strength. Electrochemical impedance spectroscopy (EIS) was utilized to determine the electrical resistivity of the cement pastes and used to infer their transport behavior. Porosity measurement was carried out using the methanol exchange method. Scanning electron microscopy (SEM) and X-ray diffraction (XRD) were conducted to gain insight into the microstructural characteristics of the cement pastes with TiO_2_ nanoparticles cured at elevated temperatures. 

## 2. Experiments

### 2.1. Materials and Specimen Preparation

**Figure 1:**
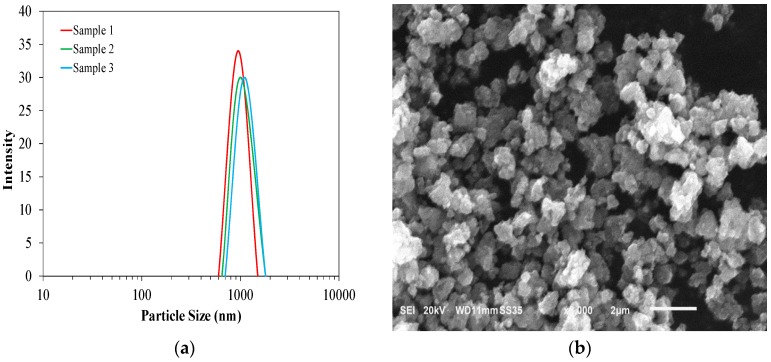
(**a**) Particle size distribution of TiO_2_ nanoparticles; (**b**) Scanning electron microscopy (SEM) image of TiO_2_ nanoparticles used in the experiment.

In this study, specimens were prepared using Type I Portland cement with the addition of 0.8%, 2.5%, and 5%, per cement mass, TiO_2_ nanoparticles. The chemical composition of the cement is presented in Table 1. The TiO_2_ nanoparticles (PC105 from CRISTAL ACTIV™, Hunt Valley, MD, USA) used in this study were the ultrafine and high purity anatase crystal phase with a surface area of about 90 m^2^/g. The particle size analysis was performed on 0.5% by mass suspension TiO_2_ nanoparticles in distilled water using dynamic light scattering with Zetasizer Nano ZS (Malvern Instruments, Malvern, UK). The particle size distribution of TiO_2_ nanoparticles is shown in Figure 1a. It is seen that the particles are in the range of 1 μm. This size is similar to those reported in the previous studies on cement paste with TiO_2_ nanoparticles [18,43]. This is due to agglomeration occurring between TiO_2_ nanoparticles. An SEM image of TiO_2_ nanoparticles is presented in Figure 1b. It should be noted that the chemical characteristics, including pH and ionic strength, of cementitious mixtures have a profound effect on the dispersion of nanoparticles and, therefore, the particle size distribution in cementitious mixtures could differ from that shown in Figure 1a. It should be emphasized that there is variation in TiO_2_ nanoparticles depending on the manufacturing processes; the TiO_2_ nanoparticles were chosen for this study on the basis of their wide use in cement-based materials, their uniform size distribution and their applications as photocatalytic material.

For cement mixture preparation, the nanoparticles were dispersed in water using ultrasonication for 30 min, and then mixed for another two minutes with a mixer before adding to the cement mixture. Cement paste cubes with 25 mm dimensions at a 0.5 water-to-cement ratio were prepared in molds of steel and glass. Neat cement pastes and the cement pastes with 0.8%, 2.5%, and 5%, per cement mass, additions of TiO_2_ nanoparticles are denoted CP, 0.8% TiO_2_, 2.5% TiO_2_, and 5% TiO_2_, respectively. The cubes were cast in two layers, with each layer tamped 32 times by a 13 mm by 25 mm tamper. After casting, the cubes were cured in a room with more than 95% relative humidity and at a temperature of 23 ± 2 °C for 24 h. The cubes were then demolded and stored in a saturated lime solution until testing.

### 2.2. Degree of Hydration

The non-evaporable water content of the cement pastes was measured at 3 days, 14 days, and 28 days of curing. This non-evaporable water content corresponds to the amount of water bound in the chemical composition of the hydration product and allows for determining the degree of hydration of cementitious materials [17,44,45,46,47,48]. About 6 g of cement paste powder was prepared by grinding and passing through the sieve #60. The powder was dried at 105 °C for 24 h and then ignited for three hours at 1050 °C. The non-evaporable water content *W*_n_ (%) was computed from the following equation:

(1) $$W_{n}=100\times \left(\frac{m_{105}-m_{1050}}{m_{1050}}-{LOI}_{c}\right)$$

where $m_{105}$ is the mass of cement paste powder after drying at 105 °C (g), $m_{1050}$ is the mass of powder after ignition at 1050 °C (g), and *LOI*_c_ is the loss on ignition of the cement estimated to be 2.03% according to the manufacturer’s specifications.

### 2.3. Compressive Strength Test

The compressive strength of the cement paste cubes was determined at 3 days, 14 days, and 28 days of curing. The compressive strength test was performed on a SATEC compression machine and the maximum compressive load sustained by each specimen was measured. The tests were conducted in stress-controlled conditions at a rate of 22 MPa/min. The average of three identical specimens was calculated and reported.

### 2.4. Electrical Resistivity Test

The electrical resistivity of the neat cement paste and the cement pastes with TiO_2_ nanoparticles cured at varied temperatures, at 3 days, 14 days, and 28 days of curing, was measured using EIS. The EIS technique avoids some difficulty prevalent in electrical resistivity measurements using a direct current (DC) arising from charge transfer resistance at the electrodes [49,50]. In this method, the imaginary and real parts of the impedance response of the cement paste cubes are measured and used to determine electrical resistivity [51]. Cement pastes cubes were sandwiched between two metallic plate electrodes. A foam, pre-soaked in a 1 M solution of NaCl, was placed between each cube surface and the electrode, and compressed by a weight to ensure a good electrical contact between the electrodes and cube surfaces. EIS measurement was conducted using a Reference Gamry 600 potentiostat/galvanostat (Gamry Instruments, Warminster, PA, USA) at an AC voltage of 250 mV and a frequency range of 10^6^–10 Hz. The electrical resistivity of the cement paste cubes was calculated as ρ *= RA/l*, where *A* and *l* are the cross sectional area and thickness of the cubes, respectively, and *R* is the electrical resistance of the cube minus the electrical resistance of two wetted foams. The average electrical resistivity of three identical cubes was calculated and reported. Cement paste cubes, cured at elevated temperatures, were allowed to cool down to room temperature before their electrical resistivity was measured. This prevented the effect of temperature on the pore solution resistivity, thereby providing an accurate measurement of the electrical resistivity of the cement pastes.

### 2.5. Porosity Measurement

Porosity measurements were carried out using small samples with a thickness of 3–5 mm obtained from the center portion of the cement paste cubes at 3 days, 14 days, and 28 days of curing. The cement paste samples were submerged in acetone to stop hydration, dried in an oven at 60 °C for two days and their dry mass measured (*m_d_*). After samples were dried, they were saturated in methanol and their mass was measured until no change in mass was observed (*m_s_*). The volume (*V*) of the samples was obtained using the Archimedes method in methanol. The porosity of the samples was measured as (*m_s_ − m_d_*)/(ρ*_methanol_V*) *×* 100, where ρ*_methano_*_l_ is methanol density. Porosity measurement was repeated for all samples and the average porosity was reported.

### 2.6. X-ray Diffraction (XRD)

The hydration product of the neat cement paste and cement paste modified with 0.8% TiO_2_ nanoparticles cured at room temperature and 60 °C was analyzed using XRD. The XRD analysis was performed on the cement pastes at 28 days of age. XRD is widely used in studying various phases in cementitious materials [7,52,53]. A small piece from the center of the cement paste cubes used in the compressive strength test was ground and passed through the sieve #60. After drying for 24 h at 105 °C in a vacuum oven, XRD was carried out using a Siemens 5000D X-ray diffractometer with the Cu Kα radiation at a scan rate of 1.5 degree/min.

### 2.7. Microscopic Examination and Elemental Analysis

Microscopic examination was conducted on the neat cement paste and the cement pastes with 0.8% TiO_2_ nanoparticles cured at room temperature and 60 °C, at 3 days and 28 days of curing. The sample preparation consisted of soaking small pieces of cement pastes in acetone to stop hydration, drying them at 60 °C for two days, and polishing them to achieve a very smooth surface finish. For polishing, samples were impregnated in a low viscosity epoxy and polished with SiC sand papers with 180, 300, 600, and 1200 grit sizes using ethanol as the lubricating medium. The samples were further polished using 1 μm polycrystalline diamond paste abrasives and cleaned in an ultrasonicator for half an hour. Then, the samples were palladium coated and examined in SEM. The Energy dispersive X-ray spectroscopy (EDS) was used to obtain the elemental composition of the hydration product in the cement pastes.

## 3. Results and Discussion

### 3.1. Degree of Hydration

**Figure 2:**
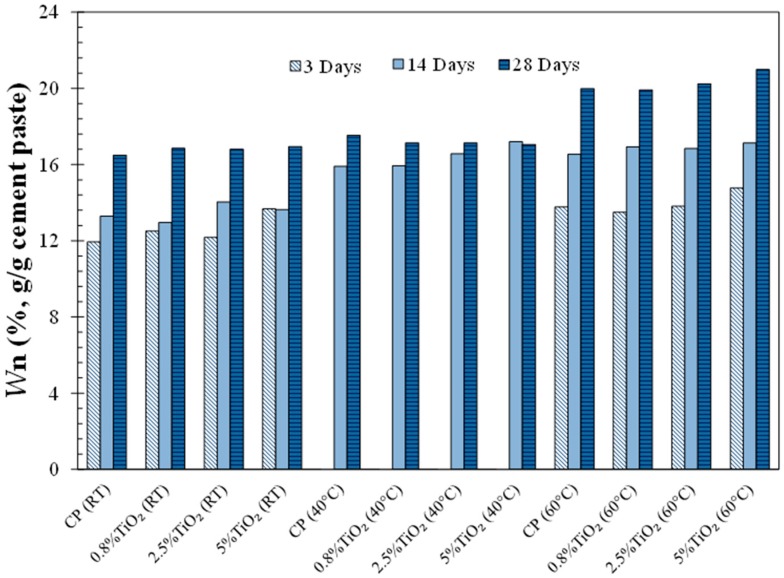
Non-evaporable water contents of the neat cement paste and the cement pastes modified with 0.8%, 2.5%, and 5% TiO_2_ nanoparticles, cured at varied temperatures (room temperature (RT), 40 °C and 60 °C), at different ages. CP: cement paste.

The non-evaporable water content (*W*_n_) of the neat cement paste (CP) and cement pastes modified with 0.8%, 2.5%, and 5% TiO_2_ nanoparticles cured at room temperature (RT), 40 °C, and 60 °C, at various ages, is shown in Figure 2. The results of curing at 40 °C corresponding to 3 days were not available due to equipment failure. It is observed that *W*_n_ increased with time in all cement pastes, as more hydration product was expected to form with time. The cement pastes cured at 60 °C showed a slightly higher degree of hydration than the cement pastes cured at room temperature at all ages. This is due to the accelerating effect of temperature on the dissolution of cement clinkers resulting in enhanced hydration at this temperature [37]. It is seen that the cement pastes cured at 40 °C experienced an increase in hydration compared to the cement paste cured at room temperature at early age, but the hydration increased at a slower rate after 14 days. The reduction in hydration rate in the cement pastes cured at 40 °C after 14 days could be attributed to the microstructure evolution. It has been suggested that at elevated temperatures, hydration product forms a dense rim around cement particles [34,41,42,54] decreasing the hydration rate of the remaining unhydrated cement particles after the initial increase in the hydration rate. It is expected that the above-mentioned microstructure evolution has also an effect on the hydration of the cement pastes cured at 60 °C; however, the increased diffusion rate through the dense rim at 60 °C could explain the observed continued increase in the hydration of these cement pastes.

It can be seen that the addition of TiO_2_ nanoparticles increased the early hydration (3 days and 14 days) of the cement pastes cured at room temperature. This is attributed to the seeding effect of TiO_2_ nanoparticles providing heterogeneous nucleation sites for hydration products [17,18]. We noted that cement pastes modified with TiO_2_ nanoparticles generally showed a similar trend at early ages at curing temperatures corresponding to room temperature, 40 °C and 60 °C. In other words, the influence of addition of TiO_2_ nanoparticles did not seem to show a distinct dependence on the temperatures used in this study. The hydration of cement is dominantly nucleation and growth controlled at the early ages and diffusion-controlled at later ages [15]. Since the effect of nanoparticles on hydration is primarily due to increased heterogeneous nucleation, it is expected that the enhancement in the hydration due to TiO_2_ nanoparticles does not depend on temperature after the first few days, where the hydration is primarily diffusion-controlled.

### 3.2. Compressive Strength

**Figure 3:**
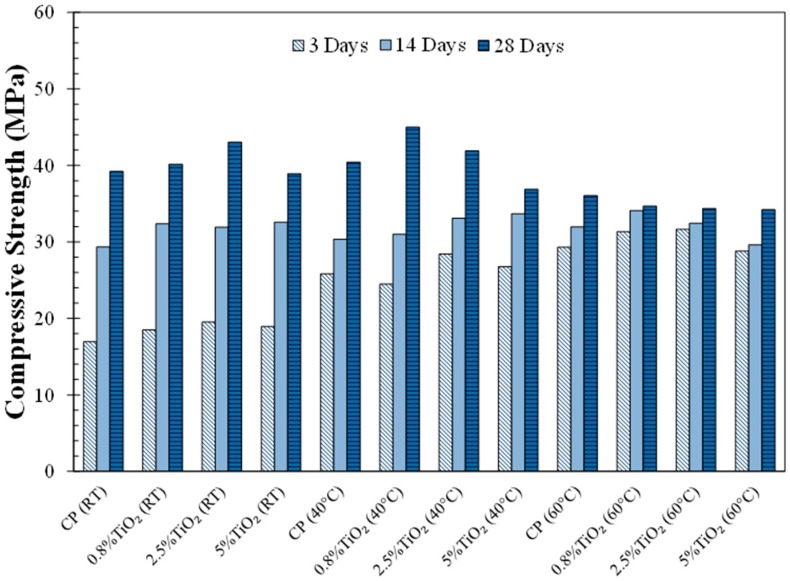
Compressive strengths of the cement pastes, cured at varied temperatures (room temperature (RT), 40 °C and 60 °C), at 3 days, 14 days, and 28 days of age.

The compressive strength of the neat cement paste and the cement pastes modified with TiO_2_ nanoparticles is shown in Figure 3. It is noted that there is an increase in the compressive strength of the cement pastes cured at elevated temperatures at 3 days, and this increase was higher at 60 °C than at 40 °C. It is noted that the cement pastes cured at 40 °C exhibited a similar compressive strength to the cement paste cured at room temperature at 14 days and 28 days of curing indicating a reduction in the strength development of these cement pastes. The cement pastes cured at 60 °C showed a slower strength gain after the initial increase at 3 days and the compressive strength of these cement pastes was lower than the cement pastes cured at room temperature at 28 days.

The observed compressive strength behavior of the cement pastes cured at elevated temperatures is attributed to the microstructural changes resulting from the hydration process at elevated temperatures. Hydration at elevated temperatures promotes rapid formation of hydration product with a non-uniform distribution in the microstructure at the early age [34]. On the other hand, hydration at lower temperatures is expected to result in a more uniform distribution of hydration product in the microstructure since the hydration process takes place at a lower rate. The increase in the non-uniform distribution of phases in the microstructure tends to increase stress concentration in the microstructure due to mechanical property mismatch, thereby degrading the overall strength of the material [54]. This will be discussed later in the paper using the SEM microscopic examination. It is expected that the effect of high temperature hydration on compressive strength depends on temperature, and most likely, the mix design and chemical composition of cement.

It is noted that the cement pastes with TiO_2_ nanoparticles showed a slight improvement in compressive strength compared to the neat cement paste at early ages (3 days and 14 days). A similar improvement in compressive strength at early ages as a result of TiO_2_ nanoparticles addition was observed in the cement paste cured at 40 °C. However, it appears that the reduction in compressive strength at 28 days in high temperature cured cement pastes is more noticeable in the cement pastes with higher additions of TiO_2_ nanoparticles. This could be due to increased heterogeneity in the distribution of hydration products in these cement pastes amplifying stress concentrations in the microstructure, thereby reducing compressive strength.

### 3.3. Electrical Resistivity

**Figure 4:**
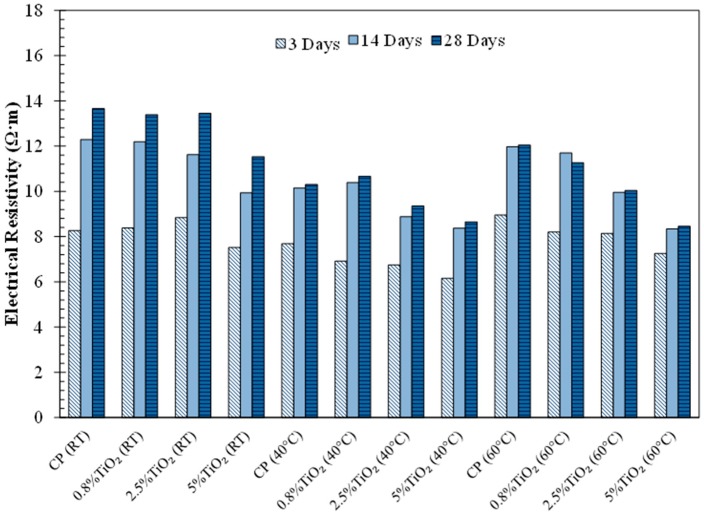
Electrical resistivity of the cement pastes, cured at varied temperatures (room temperature (RT), 40 °C and 60 °C), at 3 days, 14 days, and 28 days of age.

The electrical resistivities of the neat cement paste and the cement pastes with TiO_2_ nanoparticles are presented in Figure 4. It can be seen that the cement pastes cured at 40 and 60 °C experienced very little change in electrical resistivity between 14 days and 28 days, compared to the cement paste cured at room temperature where a gradual increase in the electrical resistivity was observed. There is a reduction in the electrical resistivity of the cement pastes cured at 40 °C compared to the room-temperature-cured cement pastes at all ages and this reduction is more pronounced at later ages. The cement pastes cured at 60 °C showed a similar electrical resistivity to the cement pastes cured at room temperature at early age, but the electrical resistivity decreased at later ages. The observed electrical resistivity results can be interpreted in view of the effect of high temperature curing on microstructure. In the cement pastes cured at 40 °C, increased hydration rate could result in a loosely packed microstructure with less dense pore structure, leading to a decrease in the electrical resistivity at all ages as seen from the figure. In the cement paste cured at 60 °C, increased hydration rate at early age was higher than in the cement pastes cured at 40 °C resulting in the production of more hydration product and offsetting the effect of heterogeneity of the microstructure, leading to a similar electrical resistivity compared to the cement paste cured at room temperature at early age. However, as seen from the figure, at 28 days, the electrical resistivity of the cement pastes cured at 60 °C was lower than that of the room temperature cured cement paste indicating the dominant effect of the loosely packed microstructure of these cement pastes on the electrical resistivity at later ages.

It is noted that the addition of TiO_2_ nanoparticles is seen to generally lower the electrical resistivity of the cement pastes and this effect is more pronounced for the 60 °C hydration. It appears that the addition of TiO_2_ nanoparticles increased the connectivity of the pore structure of the cement pastes. This is in agreement with the results of [21] but in contrast with other studies [20,32,33] where improved microstructure densification in the cement pastes modified with TiO_2_ nanoparticles was observed. The reduction in the electrical resistivity of the cement pastes with high additions of TiO_2_ nanoparticles cured at high temperatures at late ages is in agreement with the compressive strength results as shown in Figure 3, indicating a more loosely packed microstructure in these cement pastes.

### 3.4. Porosity Measurement

**Figure 5:**
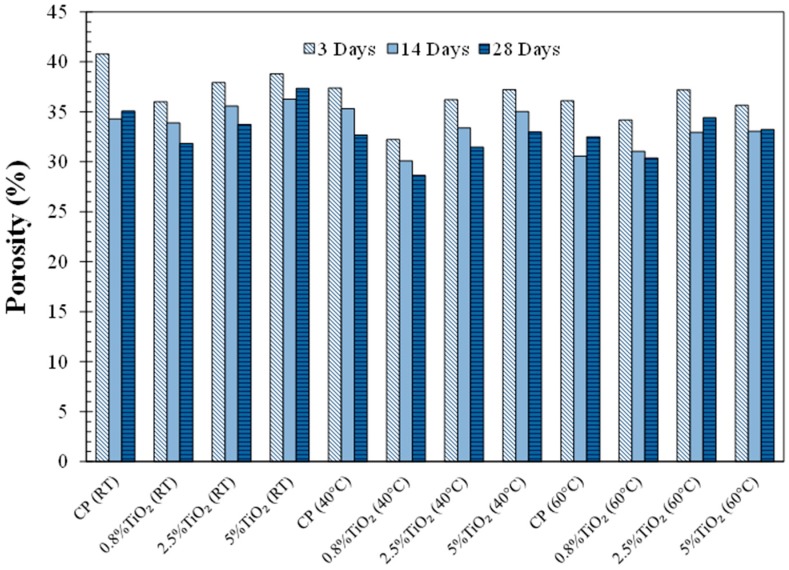
Porosity of the cement pastes, cured at varied temperatures (room temperature (RT), 40 °C, and 60 °C), at 3 days, 14 days, and 28 days of age.

The porosity results of the neat cement paste and the cement pastes modified with TiO_2_ nanoparticles cured at room temperature, 40 °C, and 60 °C are presented in Figure 5. It is notable that there is a general reduction in porosity in the cement pastes cured at 40 and 60 °C compared to the cement paste cured at room temperature. The decrease in porosity in the cement pastes cured at elevated temperatures could be attributed to an improved hydration rate at high temperatures producing more solid hydration product in the microstructure. It should be noted that the transport of cementitious material depends on the pore structure and pore solution chemistry, with the pore structure having a more pronounced role on transport behavior. In light of this, a comparison of the porosity and electrical resistivity results can be made. Porosity is a bulk material property indicating the total ratio of solid phase in the microstructure of the material. On the other hand, the electrical resistivity is highly dependent on the pore structure characteristics, such as pore size and connectivity. Thus, even though there is a reduction in porosity in the cement pastes cured at high temperatures, the electrical resistivity seemed to decrease as a result of the loosely packed microstructure of the cement pastes cured at high temperatures.

It can be seen that the addition of TiO_2_ nanoparticles was shown to generally decrease porosity at early age in most of the cement pastes, indicating improved hydration, as seen in Figure 2. However, this trend was reversed with an increase in curing time.

### 3.5. XRD Analysis

**Figure 6:**
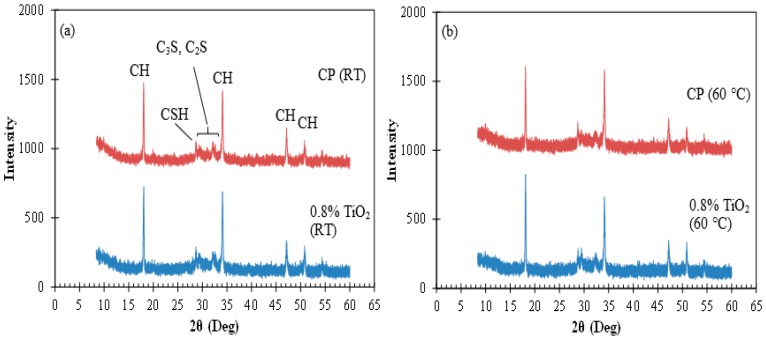
X-ray Diffraction (XRD) spectra of the neat cement paste and cement pastes with 0.8% TiO_2_ nanoparticles cured at (**a**) room temperature and (**b**) at 60 °C. CH: calcium hydroxide; CSH: calcium silicate hydrate; C_3_S: tricalcium silicate; C_2_S: dicalcium silicate.

The XRD spectra of the neat cement paste and cement paste with 0.8% TiO_2_ nanoparticles cured at room temperature and 60 °C at 28 days of age are shown in Figure 6a,b, respectively. The peaks at 2θ of 18.00°, 34.10°, 47.12°, and 50.81° correspond to calcium hydroxide [55]. It can be seen that the calcium hydroxide peaks are present in all cement pastes and exhibit a similar intensity to each other. Tricalcium silicate (C_3_S) and dicalcium silicate (C_2_S) have peaks in the 2θ range of 29°–35° [55]. C_3_S and C_2_S constitute the primary components of cement clinkers and the weak peaks corresponding to these phases in the cement pastes indicate consumption of these phases during the hydration process. There is a weak peak at 2θ of 28.68°, which can be contributed to the presence of calcium-silicate-hydrate (C-S-H) in the microstructure of the cement pastes [4]. However, it should be noted that calcium hydroxide has a peak at 2θ of 28.75° making difficult a certain identification of C-S-H at this peak. Comparing the XRD spectra of the cement pastes indicates no noticeable variation in the phases present in the microstructure of the cement pastes with and without TiO_2_ nanoparticles cured at different temperatures at 28 days of age. This indicates that high temperature curing of cement paste with TiO_2_ nanoparticles does not result in new phases in the hydration product or distinctly affect the phase composition of the hydration product.

### 3.6. Microscopic Examination

**Figure 7:**
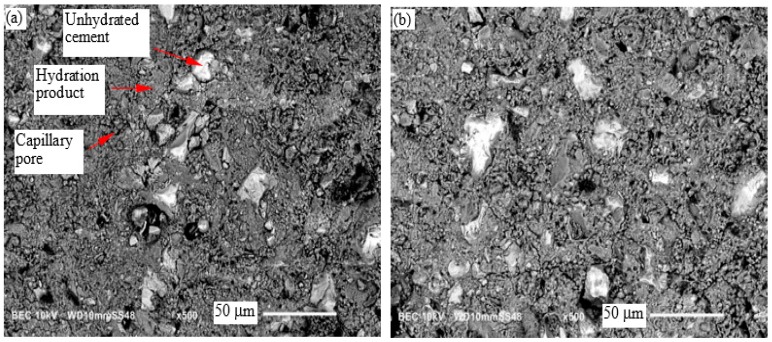

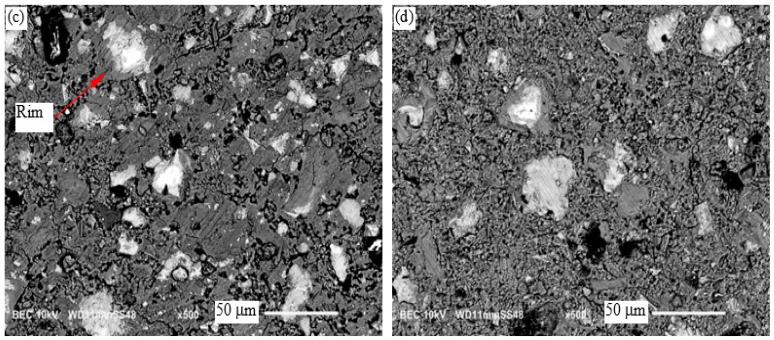
SEM micrographs showing the microstructure at 3 days of (**a**) the neat cement paste; (**b**) cement paste with 0.8% TiO_2_ nanoparticles, respectively, cured at room temperature; (**c**) the neat cement paste; and (**d**) cement paste with 0.8% TiO_2_ nanoparticles, cured at 60 °C.

**Figure 8:**
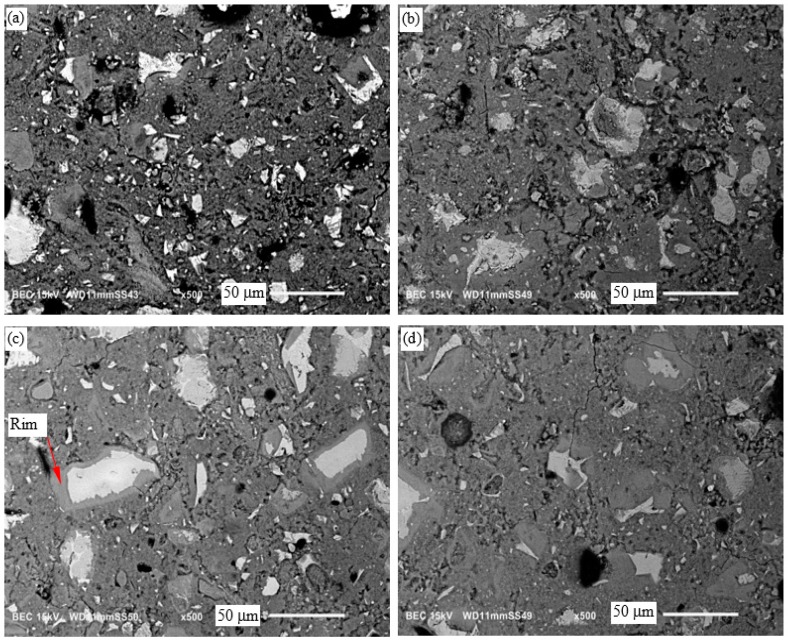
SEM micrographs showing the microstructure at 28 days of (**a**) the neat cement paste; (**b**) cement paste with 0.8% TiO_2_ nanoparticles, respectively, cured at room temperature; (**c**) the neat cement paste; and (**d**) cement paste with 0.8% TiO_2_ nanoparticles, cured at 60 °C.

The SEM micrographs of the neat cement paste and cement paste with 0.8% TiO_2_ nanoparticles cured at room temperature (RT) and 60 °C, at 3 days and 28 days of curing, are shown in Figure 7 and Figure 8, respectively. An example of an unhydrated cement particle, hydration product and capillary pores is shown in Figure 7a. A general improvement in the densification of the microstructure from 3 days to 28 days can be seen in these micrographs. A dense rim of hydration product, mostly composed of calcium-silicate-hydrate (C-S-H), is seen to have formed around cement particles in the microstructure of the cement pastes cured at 60 °C, as marked in Figure 7c and Figure 8c. The microstructure of the cement pastes cured at 60 °C consisted of dense regions and macropores dispersed in the microstructure. On the other hand, the cement paste cured at room temperature appeared to have a porous microstructure with more uniformly distributed capillary pores. The initial microstructure densification, as a result of curing at high temperature, can explain the initial increase in the compressive strength of the cement pastes, as shown in Figure 3. The increase in the heterogeneity of the phases in the microstructure of hydration product in the cement pastes cured at 60 °C increases the mismatch in mechanical properties and stress concentration at the microscale. This could explain the relatively small increase in the compressive strength of the cement pastes cured at 60 °C after 3 days, as seen in Figure 3. No significant variation in the morphological features of the cement pastes with and without TiO_2_ nanoparticles can be inferred from these micrographs cured at room temperature or 60 °C.

The results of the EDS elemental analysis of the hydration product are shown in Table 2. There appears to be a slight increase in the atomic ratio Ca/Si of hydration product cured at 60 °C compared to room temperature. On the other hand, atomic ratio Al/Ca seems to be lower at 60 °C curing than at room temperature curing. An increase in Ca/Si ratio and a decrease in Al/Ca ratio at high temperature curing has been noted in prior investigations [34,40,56,57]. A possible explanation for this observation has been suggested to be more interspersed mixing of C-S-H gels and Ca(OH)_2_ in the cement pastes cured at high temperatures [40,56]. It is noted from Table 2 that the difference in the atomic ratios Ca/Si and Al/Ca between the hydration product in the rim around cement particles and away from cement particles is insignificant, and this is in agreement with the prior studies [56]. The observed difference in the color of the hydration product in the rim and away from cement particles in the backscatter SEM images has been attributed to the difference in porosity and water content and not the chemical composition of the hydration product [56].

## 4. Conclusions

In this study, the effect of curing at elevated temperatures on the hydration, microstructure, compressive strength, and transport of cement paste modified with TiO_2_ nanoparticles was investigated. Based on the results obtained from this study, the following conclusions can be drawn:

- Temperature generally tends to increase the early hydration of the cement paste and cement pastes with TiO_2_ nanoparticles.
- It was observed that the cement pastes cured at elevated temperatures exhibited a rise in compressive strength at early age, but the rate of strength gain decreased at later ages more noticeably at a curing temperature of 60 °C than at 40 °C.
- The electrical resistivity of the cement pastes cured at elevated temperatures was shown to exhibit insignificant change after 14 days of curing. At 28 days, the electrical resistivity of these cement pastes was decreased compared to that of the cement paste cured at room temperature. It was suggested that a competition between improved hydration and non-uniform distribution of the hydration product in the microstructure appeared to determine the electrical resistivity of the cement pastes cured at elevated temperatures.
- The porosity of the cement pastes cured at elevated temperatures was shown to be generally lower than that of the cement paste cured at room temperature. The contrast between the porosity and electrical resistivity results is explained due to the dominant effect of pore structure and non-uniform distribution of hydration product on the transport behavior of cement pastes cured at elevated temperatures.
- Microstructural examination indicated a more uniformly distributed hydration product in the cement pastes cured at room temperature than in the cement pastes cured at 60 °C.
- It was observed that the effect of high temperature curing on reducing the compressive strength and electrical resistivity of the cement pastes at late ages was more pronounced with higher levels of added TiO_2_ nanoparticles.

## Figures and Tables

**Table 1:** Chemical composition of the cement.

Composition (% by Mass)	
Silica	20.82
Alumina	4.98
Iron oxide	3.68
Calcium oxide	64.34
Magnesium oxide	0.91
Sodium oxide	0.19
Potassium oxide	0.41
Sulfur trioxide	2.79
Titanium dioxide	0.24
Loss on ignition (LOI)	2.03

**Table 2:** Energy dispersive X-ray spectroscopy (EDS) elemental analysis of the hydration product. CP: cement paste. RT: room temperature.

Sample	Rim			
	Ca/Si	Al/Ca	Ca/Si	Al/Ca
CP (RT)	1.90	0.06	-	-
0.8% TiO_2_ (RT)	1.81	0.08	-	-
CP (60 °C)	1.99	0.04	1.95	0.04
0.8% TiO_2_ (60 °C)	1.97	0.05	1.89	0.04
